# Balancing the Lifetime and Storage Overhead on Error Correction for Phase Change Memory

**DOI:** 10.1371/journal.pone.0131964

**Published:** 2015-07-09

**Authors:** Ning An, Rui Wang, Yuan Gao, Hailong Yang, Depei Qian

**Affiliations:** 1 State Key Laboratory of Software Development Environment, Beihang University, Beijing, China; 2 School of Computer Science and Engineering, Beihang University, Beijing, China; Bangladesh University of Engineering and Technology, BANGLADESH

## Abstract

As DRAM is facing the scaling difficulty in terms of energy cost and reliability, some nonvolatile storage materials were proposed to be the substitute or supplement of main memory. Phase Change Memory (PCM) is one of the most promising nonvolatile memory that could be put into use in the near future. However, before becoming a qualified main memory technology, PCM should be designed reliably so that it can ensure the computer system’s stable running even when errors occur. The typical wear-out errors in PCM have been well studied, but the transient errors, that caused by high-energy particles striking on the complementary metal-oxide semiconductor (CMOS) circuit of PCM chips or by resistance drifting in multi-level cell PCM, have attracted little focus. In this paper, we propose an innovative mechanism, Local-ECC-Global-ECPs (LEGE), which addresses both soft errors and hard errors (wear-out errors) in PCM memory systems. Our idea is to deploy a local error correction code (ECC) section to every data line, which can detect and correct one-bit errors immediately, and a global error correction pointers (ECPs) buffer for the whole memory chip, which can be reloaded to correct more hard error bits. The local ECC is used to detect and correct the unknown one-bit errors, and the global ECPs buffer is used to store the corrected value of hard errors. In comparison to ECP-6, our method provides almost identical lifetimes, but reduces approximately 50% storage overhead. Moreover, our structure reduces approximately 3.55% access latency overhead by increasing 1.61% storage overhead compared to PAYG, a hard error only solution.

## Introduction

DRAM-based main memory is facing severe challenges due to its high leakage, limited scaling and increasingly refresh cost. In addition, in future 64Gb devices, almost 50% of DRAM power will be consumed by refresh operations [1]. Emerging non-volatile memory (NVRAM) is a promising technology to be the substitute or supplement of the DRAM main memory system. The phase change memory (PCM) is one of the most promising non-volatile memory for its good scalability, high density and compatibility with complementary metal-oxide semiconductor (CMOS) process.

PCM stores information by setting the phase change materials to different resistance states, which are called crystal and amorphous states respectively. The state of the material can be switched for a certain time after a strong current has passed through. The required strengths of current are different if we switch the state to different directions. Adding a tiny current to the material can detect its resistance, and then read out the stored information. This operation will not change the resistance of this PCM cell. In other words, the information is permanently stored in the PCM cells even when the electric is turned off. The tiny electric charges from the high-energy particle are far from being able to trigger the PCM memory cells to switch the state. Therefore, PCM cells have barely normal transient errors that often appear in DRAM cells.

Like other memory technologies, PCM has its own disadvantages. The average write endurance that phase change memories usually have is close to 10^8^, which is far smaller than the DRAM endurance (approximately 10^15^). If the number of writes in one PCM cell has exceeded the write endurance, this cell would be permanently stuck at fault, or worn out. With the process variation and unevenly writing, the worn out cells may appear more earlier [2]. These worn out faults are normally referred to as “hard errors”. A majority of prior works have focused on this type of errors and try to employ methods to prolong the PCM lifetime [3][4][5].

As the phase change materials have immunity to the high energy particles, conventional researches including ECP [5] and PAYG [3] seldom focus the soft errors in PCM. However, PCM also suffers soft errors. Soft errors are generally refer to the transient, undestroyed state inverses caused by the striking of high-energy particles in the storage cells or transmission lines. When transient errors occur, the device can still work. This issue has been concerned in DRAM memory, which is mainly composed by capacitors. ECC [6], and Chipkill [7] are the normal solutions. But in PCM chips, CMOS circuit also takes a big part, which also introduces the possibility of state inversion caused by high energy particles. Another issue comes from the resistance drifting in multi-level cell PCM [4][8]. We will discuss it further in section 2.

In this paper, we propose a novel method, Local-ECC-Global-ECPs (LEGE), to solve not only hard errors, but also soft errors by deploying a small local ECC, a local ECP-1 and a global ECPs buffer for each cache line size memory block(normally 512 bits). The local ECC is a single-error-correction-double-error-detection (SEC-DED) code, which can detect two errors and correct one error normally. Therefore, it can detect and correct one-bit soft error effectively in PCM, just as it is used in DRAM. As the number of soft errors in PCM is far lower than that in DRAM [4][9][10][11][12], we allocate a ECC fielded in a more large data block(512 bits) than that normally used in DRAM(64 bit). Also, note that the SEC-DED ECC can correct a one-bit hard error without having to reload the hard error data, which is one key idea in our work. The rest errors caused by worn-out cells will be corrected by a local ECP-1 field and a global ECP buffer.

Besides error corrections, LEGE also has a good tradeoff between performance and storage overhead. The benefit from our design is as follows:

- As the normally used ECC, this method can solve soft errors in the PCM circuit;
- The ECPs section in a global buffer can store corrected errors caused by worn out cells (hard errors) and decrease storage overhead;
- As the ECC is associated with the data array, it can correct one-bit error whatever the kind of the error is;
- The storage overhead of error correction is 7.1%, which is much lower than the normally used hard error correction method, such as ECP-6; this design trivially affects memory read performance.

The rest of the paper is organized as follows: Section 2 provides a background on PCM technology. Section 3 discusses the related works and motivation. Section 4 describes the framework of LEGE and the error correction methods in our work. Section 5 interprets our experimental methodology and evaluation. Section 6 concludes this paper.

## Background

### PCM Technology

A typical PCM cell consists of an NMOS access transistor and a phase change material (*Ge*_2_ *Sb*_2_ *Te*_5_). Each PCM cell has two material states with different properties: amorphous and crystalline states. When the cell is heated to its melting point and then cools down, it is in an amorphous state. In the amorphous phase, which is also called the RESET state, the cell’s resistivity is high. However, when the material is heated above its crystallization point and below its melting point, it will transform to the crystalline phase (the SET state) and have low resistivity. The PCM technology utilizes the reversible phenomena to store values. When the cell is at a low resistance state, it logically stores ‘1’. Conversely, it stores ‘0’ at a high resistance state.

Moreover, the multi-level cell (MLC) PCM has more than two states because there is a wide range of varying resistance levels in it. The MLC PCM relies on a finer-grain control in order to write accurately to expected states in a cell; it stores more data per cell by using intermediate states that are located between the crystalline and amorphous states of the phase material. Although this type of PCM can increase density by storing more information, it has a problem caused by the resistance drifting, which has been presented in some prior papers [8][9][13] in recent years. The resistance drift phenomenon is attributable to the resistance values that may increase over time. When one or more intermediate states’ resistances cross their state boundaries and the cells’ states change, PCM soft errors emerge.

### Errors in PCM

The temperature changes frequently for the storage of values. As the states change between amorphous and crystalline, the phase-change materials in PCM cells become invalid as time passes. Therefore, the lifetime of PCM decreases distinctly. Due to the process variation, the worn out phenomena in each cell occur at different times. In some cells these phenomena may emerge earlier than usual. The wear-out error problem is the topic that has received the most focus in previous research on PCM error correction.

Although phase change material is immune to the high-energy particles, the PCM memory still suffers soft errors. For example, In PCM chips, up to 40% of the whole area consists of CMOS circuits, such as row buffers, latches, and registers [14]. The data may be changed during the transmission, which caused by the high energy particles striking on CMOS. Additionally, the room temperature may cause of crystallization of phase change materials, lowering cells’ resistances beyond normal values, which is due to the unstable nature of amorphous phase [4]. Particularly, the multi-level cells may suffer from resistance drifting. They store data in different resistance levels; for example, a two-bit cell has four resistance levels (00, 01, 10 and 11). Experimental results showed that the four resistance levels of the two-bit cell drift after 400 hours at room temperature and 12 hours at 130°C [8]. While, prior work [9] has shown that four levels PCM cannot be used as main memory because of the great effect of the resistance drift.

## Related Works and Motivation

As time goes on, all PCM cells’ lifetimes decay as the frequent changes between RESET and SET states in the phase change materials. Wear-out error solutions are used to discover and correct the cells only when error cells emerge far earlier than average.

The majority of prior works have focused on cells’ wear-out problems; far fewer works pay attention to solving soft errors. Before this paper, the work paying close attention to both kinds of errors is FREE-p [4]. Besides that, the related works in the area which focus on wear-out error corrections are PAYG [3] and ECP-6 [5].

The ECC is an outstanding method used to detect and correct errors in a DRAM-based system. However, it seems less-effectively used to discover and correct wear-out errors in non-volatile memory, because these kinds of errors are dominant, irreversible errors and can accumulate.

The ECP’s aim is only focused on the PCM wear-out errors. It includes a pointer and a correction bit; the pointer can indicate the error location, and the correction bit can record the corrected data. For example, at a 512-bit memory line, ECP-n uses n entries; each entry is comprised of a 9-bit (log_2_(512)) pointer and a one-bit correction bit for one error. Therefore, ECP-n can correct n bits errors for this line. Beyond that, there is a global bit to show whether n entries are all in use. When they are all in use, the value in this bit is set to ‘1’. If they are not, it is set to ‘0’. In particular, ECP-1 uses 9 bits for storing the error location and 1 bit for correcting the error. When the system reads or writes at one line comprised of several wear-out errors, it will read or write the corrected data in ECPs instead of the wrong values in the worn out cells. This method may be effective in preventing the occurrence of a large number of wear-out errors. When hard errors in one line accumulate over time, the storage overhead of ECPs would gradually increase.

As the ECP means high overhead for memory manufactures, prior work PAYG [3] has found that ECP-6 is inefficient, and most lines do not need many error correction bits before large areas of cells become invalid. PAYG [3] proposed an attractive method for efficiently using the ECP-6 and optimizing its storage overhead, but it can only solve detectable hard errors. Their emphasis, demonstrated by their experiment, showed that more than 75% of PCM lines required no more than an ECP-1 throughout the age of ECP-6. Therefore, PAYG presented a dynamic mechanism to improve the organization of ECP: setting an ECP-1 in the local line and many ECP entries for global error correction. The global entries are used when one line appears more than one wear-out errors. This mechanism can reduce storage overhead effectively relative to the ECP-6.

Besides the wear-out errors, soft errors cannot be ignored. Both ECP and PAYG can only detect and correct hard errors. The perfect memory system must be strong in dealing with both types of errors.

FREE-p [4] is one of a few pieces of literature that addresses both soft errors and hard errors of the PCM. It uses a staged 61-bit 6-bit error correcting 7-bit error detecting (6EC-7ED) BCH code for solving both soft errors and hard errors. If even one error occurs in one line, the information in this line should be remapped and stored to a new line, but storage overhead is too expensive. When the hard errors accumulate in a data block, the BCH decoder is consequently slower. In addition, Free-p needs the help of the OS to remap the address of the disabled data block to a new area. However, this process may lead to access traffic and latency, limiting its performance. Moreover, when the page is swapped out to the disk, the OS needs to know which line is remapped. Because FREE-p lacks of a fine-grained remapping table between memory and disk.

As the PCM also faces the threat of soft errors, the existing hard error solutions cannot be put into applications unless they are as friendly as soft error solutions in normal DRAM systems. This paper provides a novel, low-cost mechanism to protect PCM-based memory against both soft errors and hard errors, while improving system reliability and prolonging cells’ lifetimes with the premise of not losing too much performance.

## LOCAL-ECC-GLOBAL-ECPS (LEGE)

Comparing with 6EC-7ED BCH [4], our method is even simpler and faster. We use a SEC-DED ECC to solve both soft and hard errors, and present a novel process that can use ECC more efficiently, since ECC was only used in DRAM in the past. We present the architecture of our work at first in this section, and then discuss the memory access process.

### Architecture of LEGE

We designed the PCM-based memory with a global ECPs buffer and a detection and correction matrix that includes valid-bits, an ECP-1 and a SEC-DED ECC.

**Fig 1:**
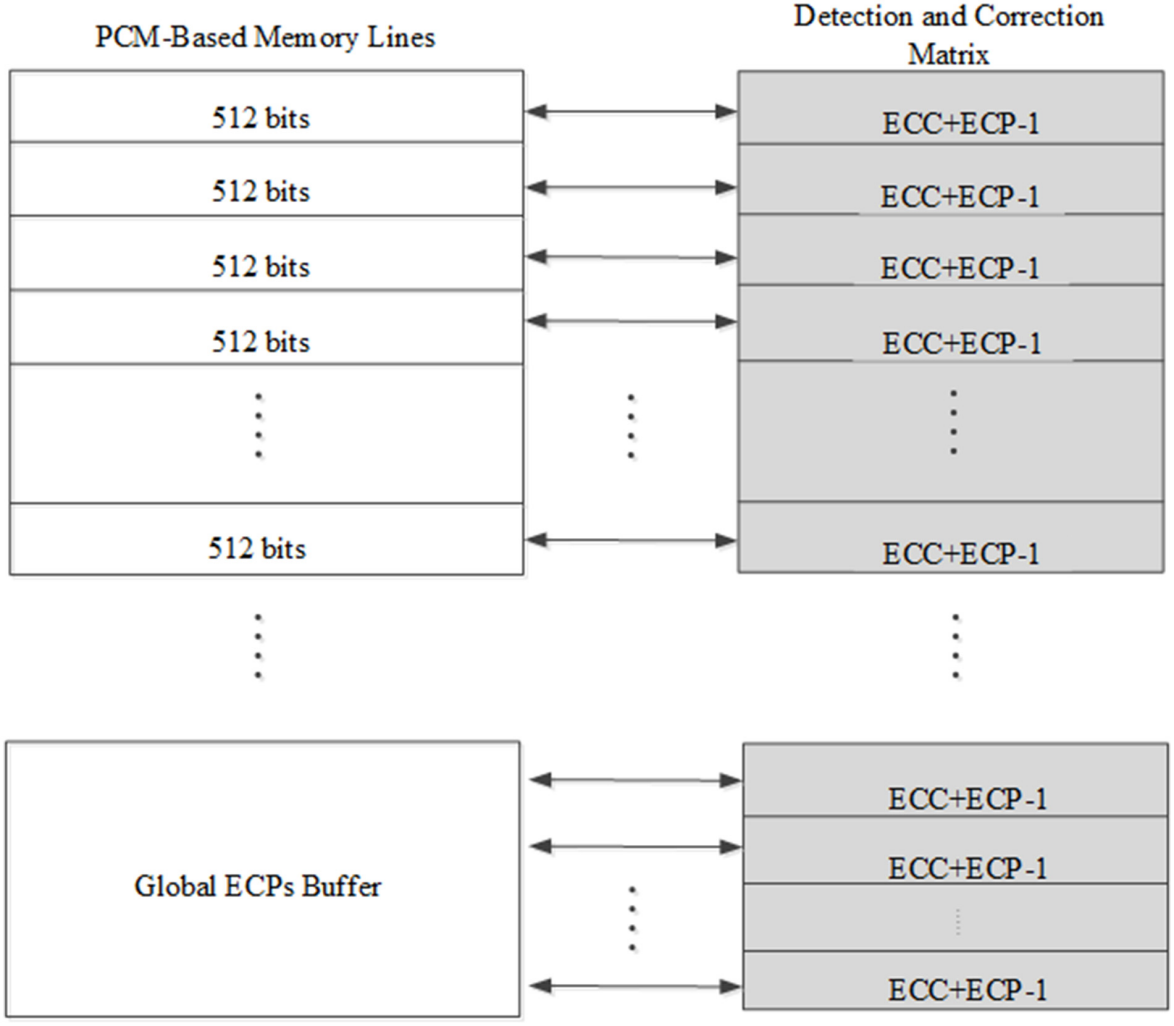
The structure of LEGE. We allocated one detection and correction matrix for one memory line. Due to the global ECPs buffer was set in memory region, each global buffer line also had a matrix for detection and correction.

Fig 1 shows the simple architecture of our scheme, which consists memory lines and an associated detection and correction matrix. Below that, there is a global ECPs buffer. We assume that one memory line can store 512-bit data, which is the same size as the last level cache line. It is just as suitable to use eight bursts incurred by a memory controller for transferring such 512-bit data.

**Fig 2:**
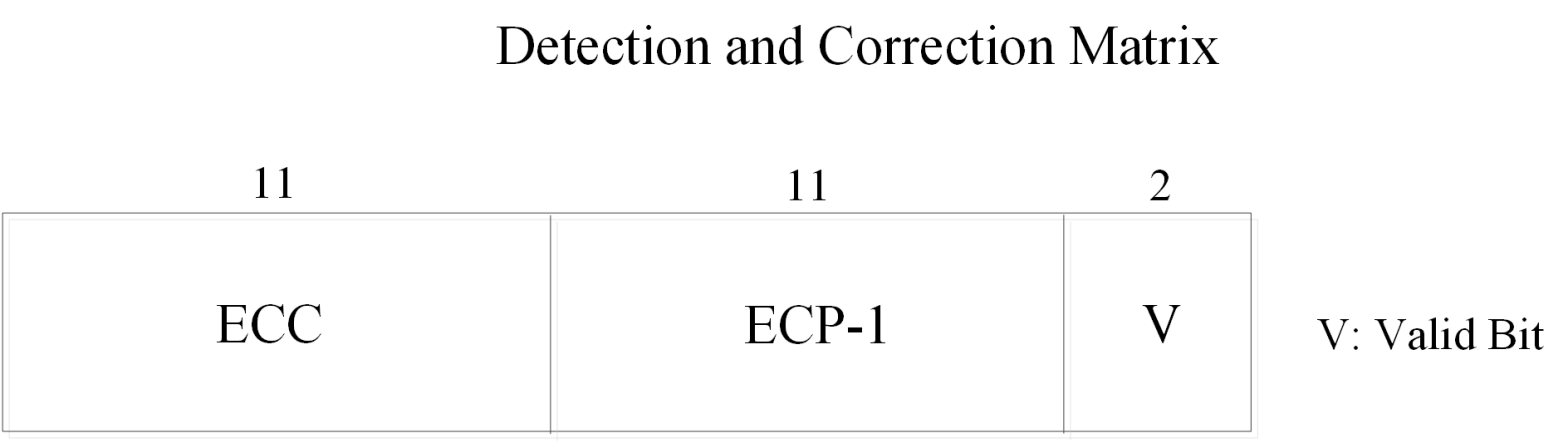
The structure of a detection and correction matrix line. Each detection and correction matrix had an 11-bit ECC, an 11-bit ECP-1 and a 2-bit valid bits.

Fig 2 presents the structure of the detection and correction matrix. One detection and correction matrix line corresponds to one memory line, which is comprised of a SEC-DED ECC, an ECP-1 and valid-bits. By setting a local ECP-1, the data lines that have one bit error don’t have to reload the correction information from global buffer. We tentatively set 24 bits for one matrix line, 11 bits suited a SEC-DED ECC.

Soft error is dominant in all kinds of errors of DRAM cells, while it is trivial in that of DRAM peripheral circuit [15]. Phase change material is almost immune to the high-energy particles which produce the DRAM cell’s soft errors [4][12]. The reliability concern of PCM is mainly caused by the resistance drift. Resistance drift was not a problem in single-level-cell (SLC) PCM which is mentioned in [4][9][10][11][12]. Because the rate of resistance drift is proportional to the initial resistance of the cell and is nearly-zero for the crystalline state [9]. The drift occurred in amorphous state does not cause error because resistance always tends to drift to a high values and all values above the boundary resistance threshold represent the amorphous state [8][11][12]. Prior work [16] has presented that the thermal interference between PCM cells at 85°C is very low even over a 10-year retention period. Moreover, as prior work [12] demonstrated that tri-level-cell (3LC) PCM has a lower soft error rate (SER) than DRAM, the SER in SLC PCM is lower than that in DRAM since boundary resistance thresholds are more closely spaced in 3LC PCM and the resistance can cross the state boundary easier than that in SLC PCM, which can lead to soft error due to state changes. So, One ECC is sufficient for a 512-bit line. We allocated 11 bits for ECP-1, which is comprised of one full bit, a 9-bit pointer and one corrected value bit. Moreover, we allocated 2 bits for valid-bit (symbol ‘V’ in Fig 2).

In valid-bit, the value ‘00’ means this memory line has no hard error so far. ‘01’ means only one hard error has occurred, so we read the corrected value from the local ECP-1. ‘10’ means there are two hard errors. ‘11’ means there are more than two hard errors. Both such situations show that the corrected data have been stored to the global ECPs buffer.

We can use three more data bits for transmitting the value in 24-bit associated detection and correction matrix during an eight-burst period; there is no need to add any more peripheral circuitry. Current server memories provide a 72-bit data path and this mechanism uses only 67 bits (64 bits for data and 3 bits for detection and correction matrix) of them.

As each local matrix can store one error, we allocated five more ECPs in global ECPs buffer for memory lines having two or more errors, which can prolong its lifetime so that it is equivalent to ECP-6. The corrected value will be put into ECP-1 or a global ECPs buffer that depends on whether this error is the first hard error. The global ECPs buffer is used to store other stuck-at-fault data bits that should be stored in worn out areas. The reloading makes the memory controller issue another memory access. These five ECPs are located in the global buffer but not in the corresponding local detection and correction matrix of each memory line. In the global buffer, there is not a one-to-one correspondence between a memory line and a five-ECPs set, which we will explain further in this section. This structure can result in a remarkable reduction of memory storage overhead.

**Fig 3:**
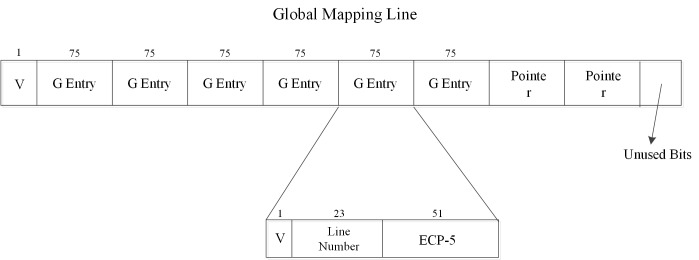
The structure of a global mapping line. Each global mapping line used a valid bit to indicate whether these six entries were all occupied. One G entry can be allocated to one memory line which had more than one error.

In Fig 3 we have provided a draft about the global mapping line in the global ECPs buffer. The architecture can provide fast access by using a Hash table structure between the memory lines and the global ECPs buffer. We assumed a global ECPs buffer line size would be equal to the memory line granularity, so it can be set into the memory region. A global mapping line includes a valid bit, six G entries and two pointers. The valid-bit indicates whether the six G entries are all occupied and whether the access operation needs one extra memory access. The value ‘1’ in the valid-bit means that all the six G entries have been allocated, and new memory line address must be mapped to a new buffer line by the pointer in this mapping line. ‘0’ means there are several G entries that are not allocated yet, and new memory line address can be mapped into this line.

Each G entry is an ECP-5 with a valid bit and a 23-bit line number. Unless this G entry is occupied by one memory line, the valid-bit in the G entry is always ‘0’. The line number section stores the corresponding memory line number, indicating in which memory line the errors occurred. The space of the line number occupied depends on the memory’s addressable space, so it is allocated with 23 bits that depends upon the memory size assumed in our work. In order to insure the pointer bits do not wear out and can function well, we used one pointer and a duplicate to protect against getting a wrong address.

**Fig 4:**
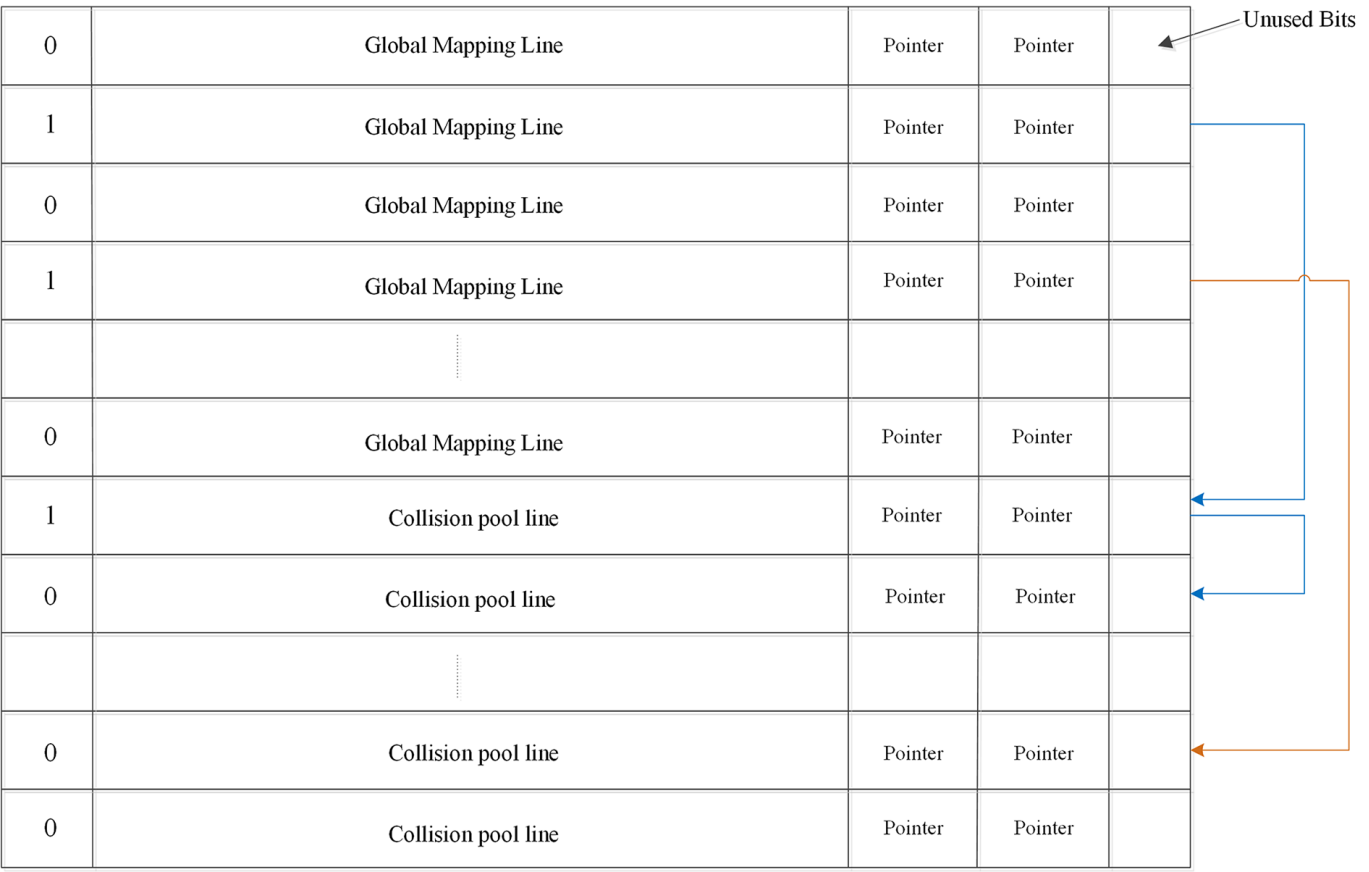
The structure of global ECPs buffer. When valid bit displayed ‘1’, it means these six G entries have already been occupied. we must use the address in pointer area to map to a collision pool line.

Fig 4 shows the simple architecture of a global ECPs buffer. The global buffer contains two parts: global mapping lines and collision pool lines. We assumed that the structure of the collision pool line is the same as the global mapping line, but they have different access patterns: the addresses of the global mapping lines must be mapped by the Hash table and the collision pool lines must be directed by the pointer in the global mapping line. The collision pool part can be flexibly deployed and only contact with the global mapping part, but not memory lines. When a global mapping line is full (the valid-bit in front of this mapping line is ‘1’), a new memory line that appears with the second error (the first error has been stored in associated local ECP-1) and is mapped into this full line should be remapped to a collision pool line by the pointer. If the valid-bit in the pointed collision pool line is ‘1’, meaning this line is already full with six entries and errors in a new memory line must be stored to another collision pool line. The pointer in this collision pool line can address a new collision line for it. The traversal continues until the valid-bit of one collision pool line is ‘0’, at which point the data can be stored in it. In the following section, we will discuss the global ECPs buffer size through experimentation.

We also deploy a detection and correction matrix to this area, like it in the memory line. When data will be written to the worn out cells in memory line, they will be written to the corresponding global buffer line. If cells in this buffer line have been written many times, they may wear out. Although the probability of occurring hard errors in corresponding global buffer line is less than it in memory line (the address bits will not be changed by rewriting and the hard error occurrence in buffer line is later than it in memory line), the reliability of global buffer cannot be ignored.

### Access Process

If the number of writes in one PCM cell has exceeded the write endurance, this cell would be permanently stuck at fault, or worn out. This phenomenon is called hard error. Verify-after-write method is widely used in write process to detect the hard error and put the corrected value into ECP.

In our work, we model the detection and correction process with considering not only the hard error but also the soft error in PCM. We use an ECC for detecting and correcting in read process. ECC can detect and correct soft error, besides that, ECC also can rapidly report the correct value of the data in hard error, but not really change the value in memory cell. The right value of data in hard error is put into ECP when it is found by verify-after-write in write process.

We explain the ECC and verify-after-write further as follow:

In the write process, we apply a widely used detection mechanism, verify-after-write. This method is successful in detecting hard errors. It writes data to one memory line and immediately reads the stored data, and then compares the written data and read data. If some bits in stored data are different from written data, it indicates that several invalid worn out bits have occurred in this line. If hard error is detected, we use one or more ECPs to record these errors (the first error will be stored in the local ECP, the others will be stored in the global ECPs buffer), and set the value of valid-bits in the detection and correction matrix. The write process can be extended by using some PCM lifetime-prolonging mechanisms, such as Flip-N-Write [17] and Coset coding [18].

In the read process, we first use SEC-DED ECC for checking memory lines; if the ECC reveals there is one error, no matter whether it is a hard error or a soft error, the ECC can immediately detect and correct it. That is the key difference in our work from prior papers. This method can largely improve performance because ECC can even correct one hard error without refetching correction information from main memory. As a majority of the memory lines have no more than a one-bit hard error in the ECP-6 service lifetime [3], our work will be effective and can maintain low storage overhead. When the error is a soft error, the local ECC corrects it directly. When the error is a hard error, the local ECC rapidly report the right data. As ECC can only report the correct values of the data in error but not really correct data by changing the memory cell values, the right value should be stored in the ECP. The hard errors can be found and corrected by the verify-after-write method in the write operation. If it is not the first one that has been found, the corrected value will be stored in the global ECPs buffer. After storing the data into the ECP, the valid-bit in the detection and correction matrix will be set automatically.

As lifetimes of PCM cells are controlled by the number of writes, the wear-out errors always occur in write operations. Our work is based on using the verify-after-write method for detecting and correcting hard errors in write operations, and using ECC for discovering and correcting soft errors in read operations. Besides that, ECC can be also used to correct wear-out errors to improve memory access performance.

Because the SEC-DED ECC can only detect two errors, when errors in one memory line exceed one, the ECC can detect but not correct it. When the errors exceed two, the ECC is unable to work. This is not suitable for PCM-based memory, because it can allow for the accumulation of errors. Combined with the use of valid-bits, the SEC-DED ECC will be available if we make small changes in the ECC circuitry. Using the ECPs’ corrected data merged ECC results (using Exclusive-OR Logic) in the ECC circuitry and letting ECC regard them as already corrected values, then the ECC can work to detect and correct soft errors. As two soft errors in a DRAM memory line scarcely occur simultaneously, the probability of two soft errors occurring at the same time can be negligible in PCM. SEC-DED ECC can be well applied in a PCM like in DRAM.

In read operations, the first step is to read the valid bits in the detection and correction matrix, and fetch the corrected data from the ECPs based on the display of valid bits. The second step is to change the ECC circuit by merging the corrected data in the ECPs, making occurred hard errors transparent to the ECC. The third step is to use the ECC for detecting and correcting the soft error.

In first step, there are four states in valid-bit:

1. If valid bits display ‘00’, there is no hard error in this line. If the ECC finds a soft error, it will correct it immediately.
2. If valid bits display ‘01’, this line has emerged only one hard error and its real value has been stored in local ECP-1. We fetch the value, and then use the ECC for detecting.
3. Valid bits display ‘10’, meaning that there are two hard errors in this line; one of the real values has been stored in the local ECP-1 and another has been stored in the global ECPs buffer. This situation is a special one. We only utilize one corrected data in the local ECP-1 rather than using both errors to change the ECC circuit. Then, if the ECC displays one error, it means that no soft error occurs in this line, and the error displayed in the ECC is another hard error for which the corrected value has been stored in the global ECPs buffer. The ECC can send the corrected value immediately without fetching it from the global buffer, which can reduce extra memory access effectively. On the other hand, if the ECC displays two errors, it means a soft error has occurred in this line. We must fetch the value in the global buffer and change the ECC circuit again (using Exclusive-OR Logic is fast), then the ECC can work to correct this soft error. The access to the global buffer in this process leads to memory access latency, but there are very few because soft errors in PCM are rare.
4. If the valid bits display ‘11’, it means that more than two hard errors have occurred. This situation indicates that we must take one extra memory access process. We need to fetch data from the local ECP-1 and the global ECPs buffer.

The changes to ECC must be ordered especially in the third state. To begin with, we must use the value in local ECP-1 to change ECC’s circuit instead of using the values from local ECP-1 and global ECPs buffer. This is a key point in our method. Because using the data from both local ECP-1 and global ECPs buffer seems easy enough, factually fetching data from global buffer can lead to at least one more memory access. Due to PCM has few soft errors, more than one memory access in the above third state rarely appears. From the above four conditions, we can see that the only conditions that will lead to taking an additional memory access are when there are two hard errors with a soft error or more than two hard errors.

In the global ECPs buffer, we allocated an ECP-5 to an associated memory line which has more than one error. Adding the local ECP-1, each memory line has six places to store corrected data. Theoretically, it can fix 7-bit hard errors with an ECC. But, we assume that it can only correct 6-bit hard errors because the ECC must be available to correct soft errors at any moment. If the ECC is used to correct the seventh hard error, and the first six hard errors have already been stored in the ECPs, the seventh right value must be sent by ECC with no place for it to be stored. At this moment, a soft error has occurred in this line; the ECC will display that there are two errors in this line and it is unable to correct any further errors. So, we assume that when the read-verify-write find the seventh hard error on it, the operating system can invalidate the physical page on the software level.

## Experiments

### Using the Distribution of Hard Errors in PAYG to Set LEGE Configuration

In order to compare with PAYG, we first use the distribution of hard errors in PAYG to set LEGE configuration. The distribution of lines with different number of hard errors tested by PAYG [3] is shown in the first line of Table 1. The distribution indicates 96.06% of lines need only one ECP, and 99.61% of lines need no more than two ECPs. Our analysis of the LEGE architecture in the previous section shows that only when there is more than one error occurring in one line will lead to extra memory access to write, so our global buffer size must be set to accommodate the hard errors in this approximately 4% of memory lines. We set the global ECPs buffer size to 64K lines, 55K lines for the global mapping portion that can store the corrected values of approximately 4% of memory lines, and 9K for the collision pool portion. We used the simple Hash function: H (k) = k% P for handling the mapping relationships, where the value of P was the largest prime number in 0 ∼ 55K-1. Our simulation generate the addresses of these memory lines by using the data of PAYG, which have more than one hard error and need access to global ECPs buffer to store corrected data at the end lifetime of ECP-6. Our experimental results show that less than 4% of memory lines need one more memory access to store data to the global mapping lines; only approximately 46000 lines need store to the collision pool once, resulting in a remaining 0.55% of all memory accesses if using our architecture. The possibility of two or more accesses to the collision pool seems negligible.

**Table 1:** The distribution of lines that using different number of ECP at the end age of ECP-6 in PAYG and our experiment.

**Num ECP per line**	**ECP-1**	**ECP-2**	**ECP-3 ∼ ECP-6**	**ECP-0**	**ECP0 + ECP1**	**ECP0 + ECP1 + ECP2**
lines/2^23^(PAYG) [3]	22.82%	3.55%	0.40%	73.24%	96.06%	99.61%
lines/2^23^(LEGE)	30%	9.2%	2.12%	58.68%	88.68%	97.88%

Based on the above analysis of ECC part in section 4, we have to go to the global buffer to fetch data only when there are more than two hard errors or two hard errors with a soft error. Due to soft errors are rare in PCM, our method can effectively reduce the access latency.

LEGE’s latency overhead (0.39%) is 3.55% less than PAYG (3.94%) throughout the lifetime of ECP-6. The storage overhead of LEGE using the data derived from PAYG is 5.5%, which is only 1.61% higher than PAYG (3.89%). LEGE used data derived from PAYG that could reduce storage overhead by 7%, compared to ECP-6(12.5%).

**Fig 5:**
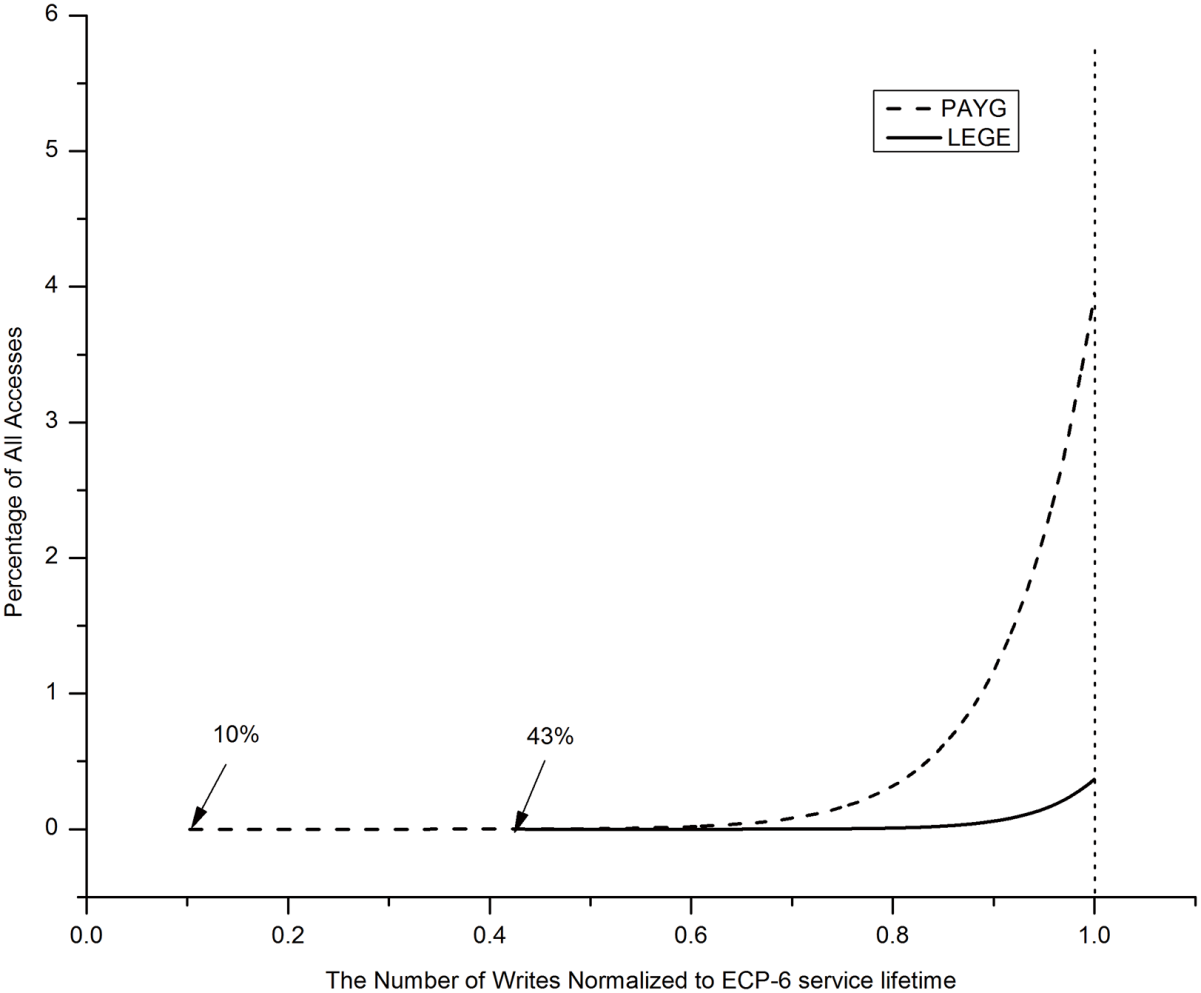
The proportion of one additional memory access among all accesses. This figure indicated that the proportion of LEGE and PAYG fetch data from global buffer among all accesses.

Fig 5 shows that one extra memory access by using PAYG architecture covers 3.94% of all memory accesses, by contrast, only incurs 0.4% in all memory accesses by using LEGE architecture at the end age of ECP-6 based PCM. The first additional access by using PAYG architecture happens at 10% of the ECP-6 lifetime, by contrast, happens at 43% of the ECP-6 lifetime by using LEGE architecture. In the first 40% of write operations, LEGE architecture will not cause any latency overhead. Moreover, LEGE only results in 0.4% latency overhead at its end age (LEGE’s lifetime is equal to ECP-6’s).

### Using the Distribution of Hard Errors in Our Experiment to Set LEGE Configuration

In order to verify the view that only a few lines require high levels of hard error correction [3]. We take an experiment similar to PAYG.

Firstly, in order to prove the accuracy of our simulation, we set the ECP-6 lifetime to 35% of theoretical maximum lifetime (each cell can be written 10^8^ and PCM can maintain 18 years in such ideal condition) with a COV of 20%, which was tested out by PAYG, to simulate the number of hard errors. After repeating the experiment several times, we found the proportion of different numbers of hard errors is almost the same as PAYG.

Secondly, we try to detect the distribution of hard errors using our own tested ECP-6 lifetime.

Memory lifetime simulation is a big challenge since it is impractical to simulate all wear patterns in a long term (PCM lifetime is more than 10 years). We use a Monte Carlo simulation to test the lifetime of ECP-6, and gather the numbers of lines using different types of ECPs. Both above two experimental configurations and assumptions are as follow:

1. We assume that the experimental memory size is 512MB with a contiguous address space. The size of the memory line is equal to the last level cache line size of 512 bits, so there are 2^23^ lines in this memory.
2. We assume that the lifetime in each cell of one PCM memory line obeyed the random Gaussian distribution which is used in prior works [3][4][5][19][20][21]. The failure bit distribution is from [21]. The mean is the average writes 10^8^[4][5] and the coefficient of variation (COV) is 20% [3].
3. We utilizes an ideal wear-leveling, which means all the memory lines will have the same number of writes, to allow our experimental results to trend normal and conservative and only focus on the error correction method impact. According to the calculation in [11] that an ECC-8 (73 bits) can reduce 7% lifetime and ECC-4 (37 bits) can reduce 4% lifetime with a wear-leveling optimization, our ECC-1 (11 bits) may only approximately reduce 1% of PCM lifetime and it is trivial.
4. The end lifetime of ECP-6 is the time that the first line fails out of all memory lines. In ECP-6, the first line fails implies that there is a data line which occurs the first uncorrectable error by deploying an ECP-6. We sort the lifetime of cells for each memory line and collect the seventh shortest cell endurance for each memory line. Then, finding out the shortest one from collection.
5. The simulation is aimed to verify the view that only a few lines require high levels of hard error correction and generate the configuration of each part according to the experimental evaluation. The soft error is rare in PCM and cannot affect the lifetime as it can be corrected by ECC immediately when it occurs. Because soft error in DRAM cell is the dominant, and it in peripheral circuit is trivial in DRAM system [15]. The soft error in PCM cell is always caused by resistance drift. The resistance drift is not a problem in single-level-cell (SLC) PCM [4][12]. So, the soft error rate in SLC PCM is far less than it in DRAM. So that, the soft error problem is not concerned in this simulation.

After repeating this experiment several times, we find the distribution of the number of failure errors is not ideal compared with it in PAYG. The detailed data in our experimental result is shown in the second line of Table 1. Table 1 shows that the distribution of lines that needs different number of ECPs according to PAYG and our experimental results. 88.68% of lines have one or no hard errors and 97.88% of lines have no more than two wear-out errors in our result. The number of no more than one error memory lines is 88.68% in our result, which is much less than 96.06% tested by PAYG. These mean that the proportion of one more access per memory access used LEGE architecture and PAYG architecture are 2.12% and 11.32% respectively, if using our experimental data. Because local area in LEGE can correct two hard errors and in PAYG can only correct one hard error. Due to the first error is stored in local ECP-1 and the excess part should be stored in global buffer, PAYG should have one more accesses to write the corrected data to corresponding global buffer. So, it gets 11.32% memory access latency if using our data.

The architecture of our work can balance storage overhead and lifetime by deploying an ECC and a local ECP-1 to reduce the number of memory accesses, and save storage overhead by setting a global ECPs buffer to store the remaining data, located in the relatively small number of lines, to decrease the associated matrix storage overhead. According to the data from the second line of Table 1, the global ECPs buffer size must be suitable in order to place lines that have more than one hard error under ECP-6 service lifetime. The size of the global mapping section we set was 192K lines, which is large enough to support 11.32% of memory lines to be mapped. We used the Hash function: *H*(*k*) = *k*%*P*, which has been proven to work well; in it, P is the largest prime number 167917 in 0 ∼ 164K-1. The result shows that less than 1.3% needs access to the collision pool portion. The size of the collision pool section we set is 28K, which is sufficient for storing data from the extra accesses. So, the pointer in the global ECPs buffer can be allocated to 15 bits.

**Fig 6:**
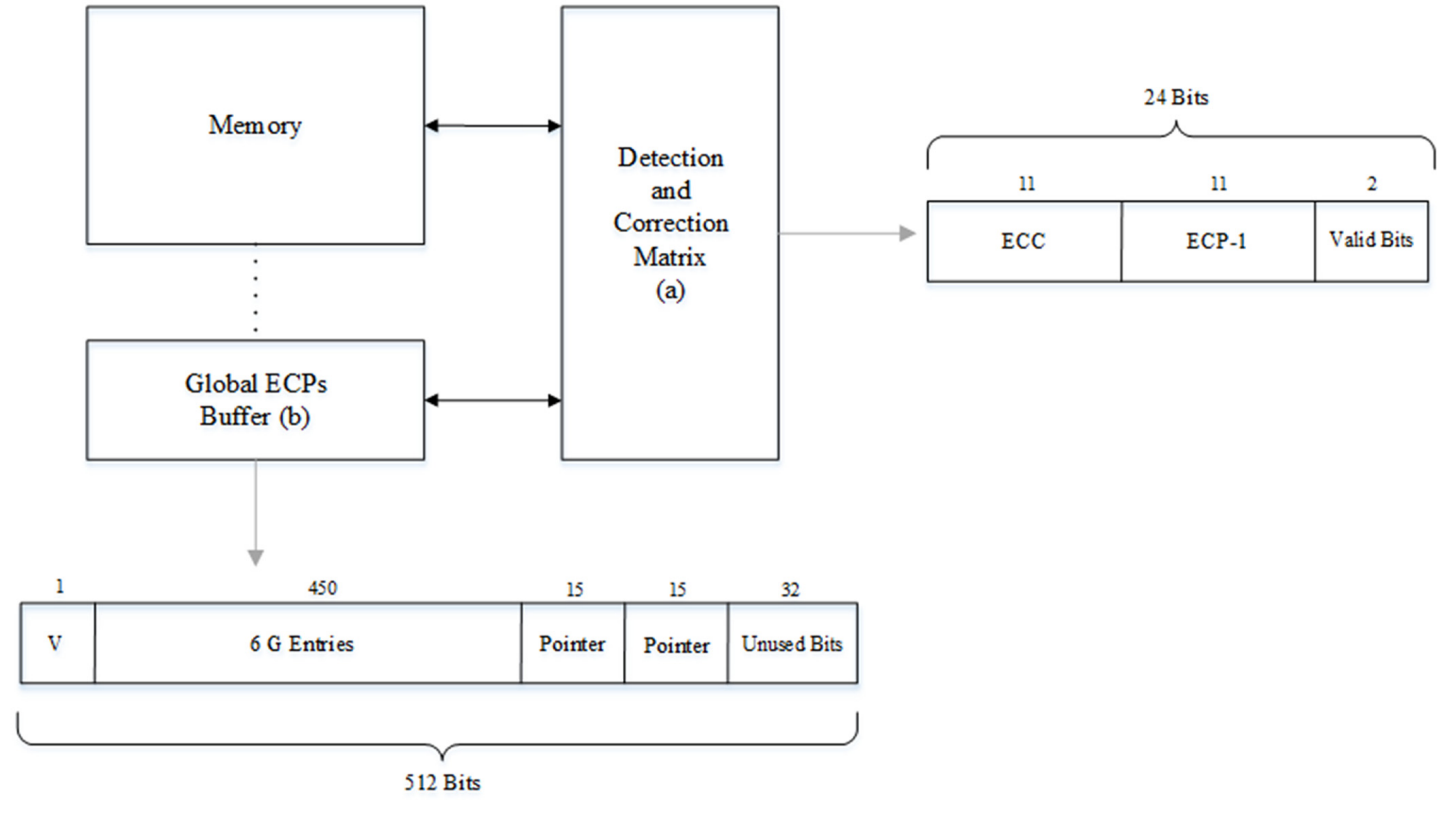
The structure of LEGE. Bits allocation for different parts in LEGE architecture.

The structure of our LEGE is presented in Fig 6. Except for the main memory, there are two parts in our framework. One is the detection and correction matrix and another is global ECPs buffer.

The calculation of detection and correction matrix’s storage overhead is as follows:

The number of detection and correction lines is 2^23^ + 192K. Where 192K is the number of these detection and correction lines that correspond to the global ECPs buffer. The total overhead of the detection and correction matrix is 24bit × (2^23^ + 192K) = 24.563MB.

The number of global ECPs buffer lines is 164K + 28K, where 164K lines is the global mapping section and 28K lines is the collision pool portion. The total overhead of global ECPs buffer is 512bit × (164K + 28K) = 12MB.

We can see the major overhead is derived from the detection and correction matrix. The total overhead of both the matrix and global buffer is 36.563MB, 7.1% of memory capacitance.

Albeit FREE-p also can correct both hard errors and soft errors, it differs from LEGE in architecture and implement method, so we cannot have a valid comparison. In FREE-p, when an error occurs in one 64B block, this block is no longer used to store data; the stored data must be remapped to a new memory line. Although FREE-p uses the other bits in this dead memory block to store the remapping pointer, the time cost in the process of remapping data cannot be ignored. Especially in Table 1, our experimental result shows that more than 40% memory lines have hard errors in the end of ECP-6 service lifetime, it means more than 40% of memory line cannot be used, we must have one more remap when these lines appear only one error by using FREE-p. However, FREE-p takes advantage in providing longer lifetime compared with LEGE. If we focus on the capacity of soft error correction, it is worth noting that comparing with FREE-p’s 6EC-7ED BCH, LEGE uses even simple and fast method to detect and correct PCM’s soft errors.

## Conclusion

In this paper, we presented a simple method for PCM-based memory reliability. This mechanism protects PCM memory against both soft errors and hard errors, provides equal lifetime for PCM chips comparing to ECP-6, and incurs only 7.1% storage overhead, which is 5.4% less than ECP-6. Comparing with PAYG, our structure can reduce approximately 3.55% latency overhead by increasing 1.61% storage overhead. Our proposal shows almost equal performance and lifetime with the hard-error-only design. This architecture is promising to handle the reliable challenges posed by PCM.
